# Neuropathic pain in athletes: basics of diagnosis and monitoring of a hidden threat

**DOI:** 10.5114/biolsport.2022.110744

**Published:** 2021-11-25

**Authors:** Yasin AlMakadma, Cristiano Eirale, Karim Chamari

**Affiliations:** 1 Aspetar, Orthopaedic and Sports Medicine Hospital, Doha, Qatar; 2 Paris Saint Germain Football Club

**Keywords:** Refractory pain, Athletes, Neuropathic pain, Nerve injuries, Complex regional pain syndrome

## Abstract

The aim was to increase awareness about neuropathic pain in athletes and the available diagnostic criteria and explore the relevance to athletes and sports. In the report of its consensus meeting of 2016, the International Olympic Committee (IOC) noted the critical need to raise the awareness about pain and its management amongst sports physicians. The adequate management of pain requires recognition of its type and pathophysiological mechanisms. This is paramount in applying the multi-modal management of pain as a symptom or approach it as a disease. In athletes, the assessment of pain in general, and of neuropathic pain in particular, is more complex due to the impact of physiological, psychological and motivational factors and specific pathophysiological mechanisms on the pain threshold and tolerance. Neuropathic pain is not uncommon to encounter in athletes although not always recognised. Examples of neuropathic pain as a disease include complex regional pain syndrome (CRPS), peripheral neuropathy and spinal cord injuries. The recognition and diagnosis of neuropathic pain could be facilitated by the application of screening tools such as DN4 (Douleur Neuropathique 4) and LANSS (Leeds Assessment of Neuropathic Symptoms and Signs). Sports injuries may lead to neuropathic pain through different pathologies and mechanisms. Thus, neuropathic pain could be a real threat to athletes’ career if not promptly recognised and treated. We therefore believe that early recognition and expert management are mandatory for the best outcome.

## INTRODUCTION

Professional athletes are typically thought of as healthy young men and women with little or no co-morbidity. Like the rest of the population, however, athletes are subject to similar communicable and non-communicable diseases. Sports practice, though, could expose athletes to painful conditions through traumatic and/or overuse mechanisms that are associated with the nature of their sports.

The medical literature offers insufficient data to tackle chronic pain (CP) in athletes from the perspective of specialised pain medicine. Indeed, pain is often a difficult area to research due to its subjective nature, the inherent complexity of factors interacting with its production and modulation, and the difficulty to objectively measure its levels [1]. In athletes, the assessment of pain is further complicated by psychological and motivational factors, and exercise-modulated thresholds and tolerance [2, 3]. This complexity extends to the choice of the best course of management of athletes suffering from chronic pain syndromes because of the low level of evidence and conflicting approaches [4, 5]. The purpose of this review is therefore to focus on neuropathic pain in athletes as it is difficult to recognise and manage. We thus aimed to provide physicians interested in sports medicine with a better understanding of the variable aspects of neuropathic pain in athletes and report simple and valid screening tools for recognition and diagnosis of this condition.

### Neuropathic pain

The International Association for the Study of Pain (IASP) defines pain as “an unpleasant sensory and emotional experience associated with, or resembling that associated with, actual or potential tissue damage”. This definition has been endorsed by the IASP task force since July 2020 [6]. From a duration point of view, the IASP defines chronic pain (CP) as that which lasts more than 3 months, which is usually a sufficient time for tissue to heal. Chronicity is an important factor in the refractoriness of the pain symptom that leads it to become a real disease on its own [7]. Included in its taxonomy, the IASP distinguishes between two essential types of pain, the *nociceptive and the neuropathic*. Nociceptive pain is produced by the activation of nociceptors, ubiquitous in the skin and inner organs. Neuropathic pain (NpP) is defined by the IASP as a *“pain caused by a lesion or disease of the somatosensory nervous system”*. Therefore, NpP pain is not a diagnosis *per se*, as it requires the identification of the causative lesion or disease. Thus, NpP is the translation of abnormal processing of the somatosensory information emitted by the body and its structures rather than the result of stimulation from the outside world [6]. Neuropathic pain is ultimately the result of an injury to the nervous system which could be caused by trauma, toxic substances or metabolic conditions that affect the neurons peripherally or centrally [8]. For sports physicians, the challenge is then to suspect and identify the type of pain reported by the athlete patient in terms of chronicity and suspected type (nociceptive, neuropathic or mixed type when both coexist) and identify physio-pathological process which caused the symptom.

In the following sections, the relevance of NpP to athletes and the diagnostic process will be discussed before giving examples of diseases, some of which are not rarely encountered in athletes.

### Diagnosing neuropathic pain

#### Relevance to athletes

NpP in athletes could be the result of direct or indirect injury to structures of the nervous system at different levels, whether central (brain and spinal cord) or peripheral (nerves and small fibres). As athletes are particularly prone to lesions of the musculoskeletal system by over-use or direct trauma, they may also be prone to injuries of the nervous system.

These could be central – for example, concussion – or peripheral, such as ulnar neuropathy [9, 10]. NpP could evolve towards refractoriness and thus become difficult to manage due to the sensitisation and complex phenomena of modulation observed at both levels of the peripheral and central nervous systems and the possible translation of specific genotypes [11, 12]. Sub-populations of athletes such as young athletes may incur a particularly heavy burden of sports-related direct or indirect nerve injuries [13]. Paralympics athletes are prone to NpP as a direct result of spinal cord injury (SCI) and/or overuse pathologies. Expert input from a pain physician is imperative in order to optimally manage not only the sport-related chronic pain syndromes but also the specific type of NpP related to spinal cord injuries [14, 15].

Moreover, as part of the general population, professional and recreational athletes are exposed to other communicable and non-communicable diseases which could be complicated by NpP as a result of viral infections, vascular and/or metabolic diseases for example.

To summarize, early recognition of NpP is as important in the athletes as it is to the general population in whom the trend of NpP towards refractoriness and chronicity is not uncommon. Physicians caring for athletes should be attentive to its signs and symptoms and have the ability to relate them to their physio-pathological mechanisms.

#### Recognising neuropathic pain

Pain is a personal experience and therefore the intensity of the reported symptoms may vary between individuals and in the same person depending on the context of injury, timing of the assessment and mechanism of the pain itself. Verbal expression of pain is just one side of each individual experience [6, 16]. It is also possible that the type of sport practised and body habitus may play a role in the perception and the pain threshold [2]. For example, refractory pain in commonly encountered tendinopathy could be due to NpP and not to the tendinopathy itself. Wilgen and Keizer reported symptoms of NpP in over 30% of athletes presenting with chronic tendinopathy [11]. Common NpP features in lower limb tendinopathy were similarly reported by Wheeler [17]. While the significance of presence of NpP in tendinopathy needs to be further investigated, it is our view that due consideration should be given for targeted treatment and specialised management of NpP in athletes with such conditions. Therefore, clinicians need first to recognize NpP by using simple and validated screening tools and scores, as we detail in the following sections.

### Screening scores for neuropathic pain

#### Douleur Neuropathique 4 (DN4)

The specificity of NpP and its potential disabling chronicity motivated the invention of scoring systems to facilitate its recognition and to timely initiate proper management. Bouhassira et al. compiled the *“Douleur Neuropathique 4”* (DN4 – French name) scoring system [18, 19]. DN4 relies essentially on the patient’s report of symptoms and on the findings of simple physical examination that may elicit an abnormal response to stimuli by impaired transmission or perception [18]. The sensitivity and specificity of DN4 are reported to be between 72% and 97% depending on the population being assessed, the language and the pathological condition being tested for NpP [22–24]. Despite its simplicity, DN4’s sensitivity and specificity are high and remain valid and reproducible when translated into different languages or applied to variable neuropathic diseases [20–22]. DN4 is based on scoring 0 or 1 for each of the 10 items of DN4. The total score thus ranges from 0 to 10. A cut-off of > 3 may indicate the presence of NpP. Despite the relative simplicity of DN4, it is expected from the clinician to be familiar with the terminology used to translate the patient’s descriptives and the findings of physical examinations. Table I shows the items of DN4 scoring criteria. The reader is also encouraged to review the definitions of terminology used in Appendix A.

**TABLE I:** Criteria used in the “Douleur Neuropathique Quatre” (DN4); Refer to main text for details.

Criteria	“Yes”	“No”
**Pain Sensation (one or more item, each score 1 or 0):** 1. Burning 2. Painful cold 3. Electrical shock	1	0
**Symptoms in the same area of pain (one or more item, each score 1 or 0):** 4. Numbness 5. Tingling 6. Pins and needles 7. Itching	1	0
**Patient’s Examination** (**one or more item, each score 1 or 0):** 8. Hypoesthesia to touch? 9. Hypoesthesia to pinprick? 10. Pain provoked or increased by brushing?	1	0
Total Score (maximal)	10	0

#### Leeds Assessment of Neuropathic Symptoms and Signs

Bennet suggested the application of the Leeds Assessment of Neuropathic. Symptoms and Signs (LANSS) as a screening tool for NpP [25]. This scoring system relies on both reported and provoked symptoms of abnormal transmission and/or perception of neurostimuli. As its name suggests, the LANSS does not exclusively focus on pain but also on “neuropathic symptoms”. The score’s cut-off value is 12 with a sensitivity often reported between 70% and 80% and a specificity reported by some authors being as high as 100% [23, 24]. While the LANSS could be considered as an effective tool to use in the context of screening for NpP, it may be viewed as more complex and may require a slightly longer time to complete compared to DN4.

The *S-LANSS* is a self-administered version of the LANSS which may serve as a screening tool for NpP in athletes while waiting for their consultation as it requires minimal guidance. It lends itself similarly well for this purpose when translated into other languages [26, 27]. Table II shows the items used in the self-administered LANSS scoring system.

**TABLE II:** Criteria used for the S-LANSS; Refer to main text for details

Item	“Yes” score	“No” score	Maximal score
In the area where you have pain, do you also have “pins and needles”, tingling or prickling sensations?	5	0	5
Does the painful area change colour (perhaps look mottled or more red) when the pain is particularly bad?	5	0	5
Does your pain make the affected skin abnormally sensitive to touch? Getting unpleasant sensations or pain when lightly stroking the skin might describe this	3	0	3
Does your pain come on suddenly and in bursts for no apparent reason when you are completely still? Words like “electric shocks”, jumping and bursting might describe this	2	0	2
In the area where you have pain, does your skin feel unusually hot like a burning pain?	1	0	1
Gently rub the painful area with your index finger and then rub a non-painful area (for example, an area of skin further away or on the opposite side from the painful area). How does this rubbing feel in the painful area?	Pins and needles, tingling, electrical shock = 5	same as painful area = 0	5
Gently press on the painful area with your fingertip and then gently press in the same way onto a non-painful area (the same non-painful area that you chose in the last question). How does this feel in the painful area?	Numbness or tenderness = 3	same as painful area = 0	3
**Total maximal score**	**24**	**0**	**24**

### Other tools

Other screening tools, such as the neuropathic pain questionnaire (NPQ) and “Pain-Detect” scores, are also available for screening of NpP. These are less frequently used in clinical practice. The reader is advised to consult additional literature such as the articles of Wheeler, Vaegter and Krauz for further details [17, 28, 29].

### Monitoring of neuropathic pain

The progress of NpP and eventual response to variable modes of management need to be monitored in order to adapt it to the evolving disease in terms of medications or procedures indicated depending on the type and mechanism of NpP. The timing and extent of return to play require regular evaluation depending on successful management of the disease. There are no objective tools for monitoring the progress of NpP or its response to management. For instance, neither DN4 nor LANSS is intended as a monitoring tool.

Physicians continue to rely on simple pain scores [Numerical Rating Score (NRS) and Visual Analogue Score (VAS)] to evaluate the clinical status of NpP and its responsiveness to management. It is the authors’ view that such an approach may be insufficient. The NRS, for instance, would only reflect the perceived intensity of pain without any reference to its type, its impact on other aspects of the patient’s life, or the impact on the athlete’s performance. It is also our view that the impact of NpP on the athlete’s performance has to be specifically evaluated through the development of new scoring systems that should integrate quantitative pain testing methods and the self-reported symptoms and signs of NpP. Patient’s Report of Outcome Measures (PROMs) represent an important tool used for the evaluation and monitoring of impacts of different types of health issues and diseases on the patient’s health [30, 31]. PROM criteria are variable in types and weight in the assessment of the patient condition. In addition, not all these PROMS can be universally applied. In our own experience, we find the application of the EuroQoL of 5 dimensions (EQ-5D) practical as it includes items pertinent to the case of athletes – that could be combined with NRS and DN4 [32, 33]. EQ-5D records the following five items: mobility, daily activities, self-care, pain-discomfort, and anxiety-depression. The impact of a given disease on these dimensions is graded as: normal (no impact), slight, moderate, severe or extreme (disabling). Patients could provide a form of their PROMs before the consultation allowing physicians to incorporate valuable information in the assessment of the patient’s condition.

### Examples of neuropathic pain

#### Complex regional pain syndrome (CRPS)

The exact pathophysiology of CRPS remains obscure [34]. CRPS is more prevalent in females and is reported in as many as 10 to 35% of patients following hand trauma requiring surgical interventions [35]. CRPS may result from various minor or major trauma, surgical insults or discopathy [36, 34]. CRPS could also affect adolescents and even paediatric subjects [37]. Classically, the disease is said to be of “Type I” when not linked to nerve injury or of “Type II” when a specific nerve injury is identified [38]. CRPS is characterized by a usually severe NpP (allodynia and hyperalgesia - See Appendix A for definitions), with vascular autonomic and trophic dysfunctions. Vascular dysfunction includes oedema, skin discolouration, abnormal skin temperature and impaired sweat function. Trophic signs include abnormal hair growth in the affected region and bone demineralization, and it may cause retracted tendons and muscles in inadequately managed cases [39].

As consequences of CRPS, impaired motor function, of both the ipsilateral and contralateral limbs, retracted muscles and tendons and “neglect syndrome” of the affected limb are examples of how a sports career could be severely compromised [40]. The early recognition of the disease is critical for the best outcome. Specialized management requires a high degree of expertise and multi-disciplinary input and sustained rehabilitation programmes [41]. In paediatric and adolescent cases, an in-depth analysis of the psychological family context should be actively considered so to adequately involve the family in the care plan [42].

#### Nerve roots and peripheral nerve injuries

In their report of 346 cases of athletes referred with sports-related injuries, Krivickas et al. reviewed the electrodiagnostic studies performed in 216 cases. The authors were able to identify various neuropathic injuries. Lesions of nerve roots, plexus or peripheral nerve injuries were identified in 180 cases. Upper limbs were more affected by neuropathic injuries than the lower limbs [43, 44]. This significant prevalence confirms the importance of recognition of NpP by sports physicians.

Suprascapular neuropathy (SSNp), which has received much of interest over the past few years, is a good example of the importance of recognition of NpP. Shoulder pain is a common complaint among athletes of various sports such as football, volleyball, handball and weight lifting [45]. Shoulder pathology encountered in wheelchair athletes represents more specific entities [15]. Athletes may present with refractory symptoms including pain, muscular hypotrophy, and impaired muscular function or proprioception of the shoulder due to SSNp [46]. Refractoriness of shoulder pain and poor response to conventional management should raise the question of the neuropathic mechanism and steer the attention towards neuropathic pain. Entrapment of the suprascapular nerve (SSN) could occur at the level of the suprascapular notch or the spinoglenoid notch. Good outcomes are reported with conservative management of SSNp [47]. The indication for surgical decompression of SSNp is however controversial [5, 48]. The outcome of surgical decompression is likely to be affected by the timing of intervention and hence the extent of SSN lesion, which could explain the controversy about its indication.

#### Spinal cord injuries

Complex mechanisms lead to NpP in spinal cord injuries (SCI) [49]. The consequences of SCI are not limited to loss of muscular power, but also include neuropathic pain, sensory loss, disturbed proprioception and osteoporosis. Detecting NpP is therefore crucial in the management of wheelchair athletes who have suffered SCI. The management of NpP and its associated symptoms of SCI requires a complex integration of pharmacotherapy, interventional pain therapy, and possibly the use of implantable devices such as spinal cord stimulators. These methods can only be successful if applied within a multidisciplinary environment managed by committed professionals [50].

## CONCLUSIONS

Pain is a common issue in athletes and results from a range of conditions varying from simple overuse to severe trauma. In addition to recognizing the cause, it is imperative to correctly diagnose the type of pain in question. As neuropathic pain is a real challenge to diagnose and treat, sports physicians should familiarise themselves with its characteristics and the available therapeutic options, which are not limited to the prescription of analgesics. Specialised techniques and pharmacotherapy can be of real help in facilitating rehabilitation and return to play of the athletes once neuropathic pain has been diagnosed and monitored.

The authors strongly believe that neuropathic pain in athletes warrants specific research investigating the methods of screening, diagnosis, evaluation and treatments. Awareness of neuropathic pain signs and symptoms may well contribute to early diagnosis and management in cases where return to play is delayed and recovery is slow despite usually adequate rehabilitation and management. Monitoring tools of neuropathic pain need to be developed and validated in order to track the patient’s recovery and time to return to play in athletes.

## Conflict of interest declaration

All authors declare having no conflict of interest.

## APPENDIX A

### Definitions quoted from IASP Taxonomy

– **Allodynia** “Pain due to a stimulus that does not normally provoke pain.”
– **Analgesia** “Absence of pain in response to stimulation which would normally be painful.”
– **Hyperpathy** “A painful syndrome characterised by an abnormally painful reaction to a stimulus, especially a repetitive stimulus, as well as an increased threshold.”
– **Hyperalgesia** “Increased pain from a stimulus that normally provokes pain.”
– **Hypoesthesia** “Decreased sensitivity to stimulation, excluding the special senses.”
– **Causalgia** “A syndrome of sustained burning pain, allodynia, and hyperpathia after a traumatic nerve lesion, often combined with vasomotor and sudomotor dysfunction and later trophic changes.”
– **Neuropathy** “A disturbance of function or pathological change in a nerve: in one nerve, mononeuropathy; in several nerves, mononeuropathy multiplex; if diffuse and bilateral, polyneuropathy.”
